# Polyethylene Terephthalate Micro and Nanoplastics Induce Oxidative and Endoplasmic Reticulum Stress, Compromising Intestinal Epithelial Integrity and Viability

**DOI:** 10.1111/all.70291

**Published:** 2026-03-25

**Authors:** Sena Ardicli, Huseyn Babayev, Lihong Chang, Ozge Ardicli, Can Zeyneloglu, Yagiz Pat, Duygu Yazici, Xiaoqing Liu, Ezgi Irem Bektas, Carina Beha, Halil I. Akyildiz, Nazrin B. Mammadova, Muhammed Emin Sari, Beate Rückert, Raja Dhir, Ismail Ogulur, Kari C. Nadeau, Mubeccel Akdis, Cezmi A. Akdis

**Affiliations:** ^1^ Swiss Institute of Allergy and Asthma Research (SIAF) University of Zurich Davos Switzerland; ^2^ Department of Genetics, Faculty of Veterinary Medicine Bursa Uludag University Bursa Turkiye; ^3^ Division of Food Processing, Milk and Dairy Products Technology Program, Karacabey Vocational School Bursa Uludag University Bursa Turkiye; ^4^ AO Research Institute Davos Switzerland; ^5^ Department of Textile Engineering, Faculty of Engineering Bursa Uludag University Bursa Turkiye; ^6^ Department of Physics, Faculty of Science Selcuk University Konya Turkiye; ^7^ Department of Medical Biology, Faculty of Medicine Necmettin Erbakan University Konya Turkiye; ^8^ SEED Inc. Co. California Los Angeles USA; ^9^ Department of Environmental Health Harvard T.H. Chan School of Public Health Boston Massachusetts USA

**Keywords:** barrier, epithelium, inflammation

To the Editor,

Plastic pollution constitutes an escalating global crisis and poses serious threats to ecosystems, human and animal health, food, water, and economies. An expanding body of evidence indicates that micro‐ and nanoplastics cannot be controlled by biological tissue barriers; they can circulate through tissues, reach distant organs, and readily accumulate within various biological systems [1, 2, 3, 4, 5, 6]. Among the many types of plastics, polyethylene terephthalate (PET) is one of the most common polymers exposed in daily human and animal life. Notably, PET fragments into micro‐ and nanoparticles that have sparked significant interest in potential health implications upon ingestion. In the present study, we investigated the effects of PET microplastics on human intestinal cell cultures subjected to a range of concentrations (~200; 1000; 10,000; 20,000; and 72,000 particles/mL) of PET particles (quantified size range ~6–8 μm, containing a polydisperse mixture of micro‐ and nanoplastics). The chemical fingerprint and ultrastructural characteristics of the PET microplastics were confirmed by Raman spectroscopy and scanning electron microscopy (SEM) analysis (Figure S1). Integrated SEM micrographs (Figure S1C–D) revealed irregularly shaped micro‐ to nanoscale PET particles with measured dimensions around ~213–217 nm, while Raman spectral analysis (Figure S1F,G) displayed distinct characteristic peaks across the 600–1700 cm^−1^ region (Figure S1G), together confirming both the nanoscale morphology and molecular identity of the detected particles and providing direct structural–chemical validation of their presence in the samples. Our findings demonstrate a toxicological cascade in which PET microplastics compromise intestinal barrier function and perturb cellular homeostasis, with oxidative stress, endoplasmic reticulum (ER) stress, and cell death emerging as principal intermediaries in the affected tissue disruptions.

In our model of exposure, namely gut‐on‐a‐chip, a pivotal metric in our investigation was the transepithelial electrical resistance (TEER) assay, which quantitatively assesses the integrity of the epithelial barrier. Remarkably, increasing PET microplastic concentrations resulted in a clear, dose‐ and time‐dependent reduction in TEER (Figure 1A). Concurrently, we employed the 3‐(4,5‐dimethylthiazol‐2‐yl)‐2,5‐diphenyltetrazolium bromide (MTT) assay to evaluate metabolic activity as an indicator of overall cellular viability (Figure 1B). While minimal toxicity was observed at lower concentrations, the higher doses (20,000 particles/mL and above) induced considerable cell death on day four. Notably, TEER reduction occurred at lower doses (1000 p/mL) prior to significant cytotoxicity, suggesting specific barrier disruption distinct from cell death.

**FIGURE 1:**
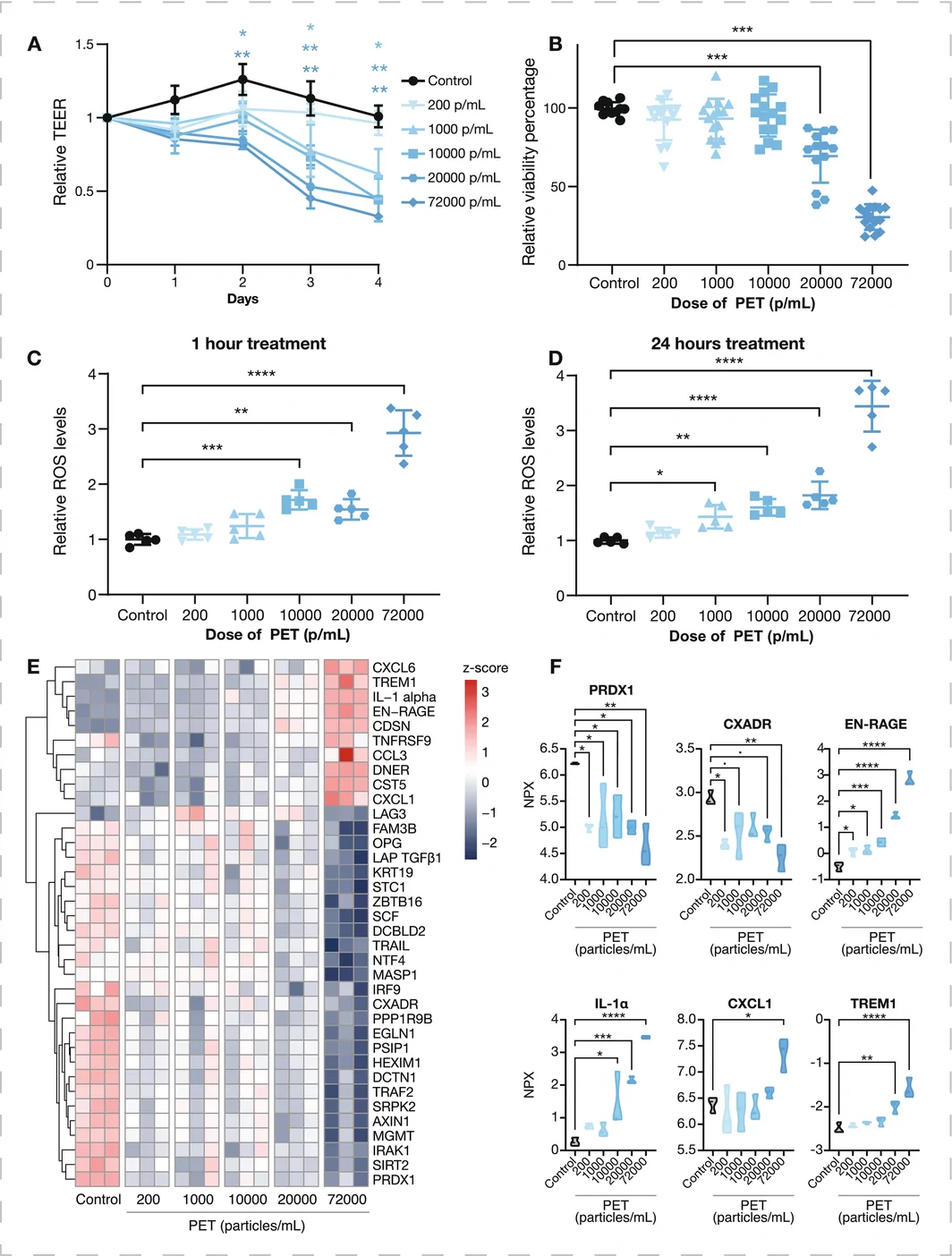
In vitro outcomes of PET microplastic exposure on human intestinal cells. (A) This graph depicts the relative change of transepithelial electrical resistance (TEER) over four days. It demonstrates a dose‐ and time‐dependent decrease in epithelial barrier function, with the most significant reductions occurring at concentrations of 20,000 and 72,000 particles/mL. (B) Relative viability percentage of cells after exposure to various doses of PET microplastics. A notable, dose‐dependent decrease in cell viability is observed, particularly at doses of 20,000 and 72,000 particles/mL. (C,D) Relative fluorescence intensity from the DCFDA assay, a measure of intracellular reactive oxygen species (ROS), after 1‐h (C) and 24‐h (D) treatments. Both show a significant, dose‐dependent increase in ROS, indicating oxidative stress. Statistical comparisons were performed using one‐way ANOVA followed by Dunnett's test. Significance levels: *p < 0.05, **p < 0.01, ***p < 0.001, ****p < 0.0001. (E) The heatmap illustrates the relative abundance of secreted proteins in the basal culture medium, as profiled by targeted proteomics. The clustering reveals a distinct, dose‐dependent pattern based on PET concentration. The profiles of samples exposed to higher doses of PET microplastics are clearly distinct from the control and low‐dose groups, indicating a significant alteration in the cellular secretome. (F) Dose‐dependent increases in proteins, indicating an inflammatory response, including the danger signals EN‐RAGE and IL‐1α, and the neutrophil‐recruiting chemokine CXCL1. Downregulation of the antioxidant enzyme PRDX1 indicates a decreased protection against oxidative stress, while changes in the tight junction protein CXADR suggest compromised barrier integrity.

With the aim of elucidating the underlying mechanisms, we first measured intracellular reactive oxygen species (ROS) generation. A marked increase in ROS production paralleled increasing concentrations of PET microplastics, even in the first hour of treatment (Figure 1C), and this effect persisted for 24 h (Figures 1D and S1B). Beyond these intracellular stress markers, we sought to determine whether this internal state prompted a functional response, as reflected in secreted proteins. By analyzing culture supernatants with proximity extension assay‐targeted proteomics, we profiled the cellular secretome and identified profound, dose‐dependent alterations. A heatmap of the proteomic data shows a distinct clustering pattern, separating the high‐dose exposure groups from the control and low‐dose groups (Figure 1E). Relatively low doses of micro‐ and nanoplastics, such as 200 particles/mL, which are relevant to daily exposure [2, 3], show significant effects on levels of many proteins (Figures 1F and S2). This protein‐level data shows that the antioxidant enzyme peroxiredoxin (PRDX1) and the junctional protein coxsackievirus and adenovirus receptor (CXADR) are significantly downregulated, while the danger signal extracellular newly identified RAGE‐binding protein (EN‐RAGE) and key inflammatory mediators IL‐1α, C‐X‐C motif chemokine ligand 1 (CXCL1), and triggering receptor expressed on myeloid cells 1 (TREM1) are significantly upregulated in a dose‐dependent manner (Figure 1F).

The dose‐dependent physiological and proteomic changes were demonstrated by an intense transcriptional change, as observed in RNA sequencing of the exposed epithelial cells. Evaluations of global gene expression, including principal component analysis (PCA) (Figure 2A) and a comprehensive heatmap of differentially expressed genes (Figure 2B), revealed distinct clustering patterns that reflected the PET microplastic dose. To investigate the functional consequences of these gene expression alterations, gene ontology (GO) enrichment analysis revealed that upregulated genes were significantly enriched in pathways related to apoptosis, endoplasmic reticulum (ER) stress, and cellular responses to oxidative stress (Figure 2C). Detailed heatmaps for these key pathways illustrate a clear particle‐number‐dependent upregulation of genes involved in oxidative stress (Figure 2D), chemical stress (Figure 2E), ER stress (Figure 2F), and the apoptotic signaling pathway (Figure 2G). Low‐dose PET microplastic exposure elicited minimal transcriptional perturbations, consistent with adaptive homeostatic responses, whereas high‐dose exposure triggered widespread pathway dysregulation indicative of oxidative stress and metabolic reprogramming (Figure S3).

**FIGURE 2:**
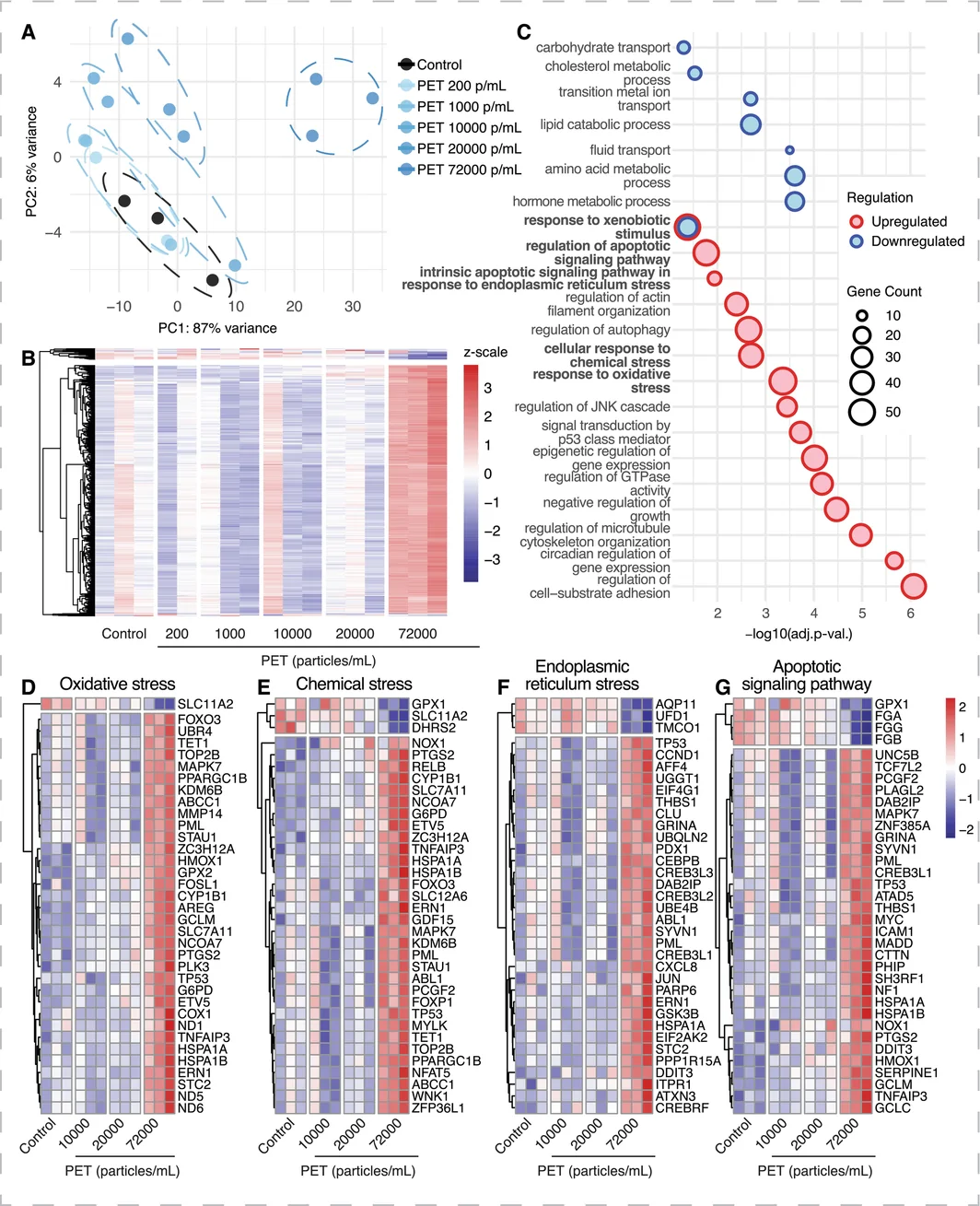
Transcriptomic characterization and pathway analyses of PET‐exposed intestinal cells. (A) Principal component analysis (PCA) plot of global gene expression shows a clear separation of samples based on the dose of PET microplastics. The low‐dose and control groups cluster on the left, while the high‐dose groups cluster on the right, along PC1, which accounts for 87% of the variance. (B) A comprehensive heatmap that captures the relative expression of differentially expressed genes across all tested concentrations. It reveals distinct clusters and widespread changes in gene expression at higher doses of microplastics. (C) Gene ontology (GO) enrichment bubble plot identifies the biological processes most affected by PET exposure. It highlights a significant upregulation (red circles) of pathways related to oxidative, chemical, and endoplasmic reticulum stress, as well as apoptotic signaling. Downregulated processes (blue circles) are primarily related to metabolism and transport. Heatmaps provide a detailed view of gene expression changes within specific key pathways. They illustrate a progressive, particle number‐dependent mostly up‐ but also down‐regulation of genes involved in (D) oxidative stress, (E) chemical stress, (F) endoplasmic reticulum stress, and (G) apoptotic signaling pathway.

This widespread transcriptional reprogramming towards stress and apoptosis provides a mechanistic basis for the ultimate decline in cellular function and health. In this context, environmental factors, including micro‐ and nanoplastics, contribute to the disruption of the epithelial barrier, which plays a critical role in the pathogenesis of various diseases [7]. This study clearly demonstrates, for the first time, that the micro‐ and nanoplastic burden of PET at daily exposure levels compromises both barrier integrity and core metabolic capacity, which could impair nutrient absorption and immune defenses, particularly under chronic or high‐level exposure scenarios. Given the pervasive presence of PET in the everyday environments of both humans and animals, the findings highlight the potentially serious consequences of PET exposure and emphasize the urgency of further investigation into its regulatory and health impacts [2, 3].

Overall, our results delineate the multifaceted nature of microplastic‐induced cellular damage, represented by diminished epithelial barrier function, decreased cell viability, and widespread transcriptional shifts converging on oxidative stress, ER stress, and programmed cell death pathways. These in vitro observations contribute to the body of evidence concerning the potential molecular mechanisms of microplastic ingestion on human health. The findings support the continued evaluation and scrutiny of permissible microplastic thresholds in food and water, particularly in the context of increasing global plastic production. Additional in vivo and dose–response studies will be critical for extrapolating these findings and refining our understanding of how microplastics manifest toxicity within the human gastrointestinal tract. Ultimately, our findings illustrate the sensitivity of the intestinal epithelium as a primary target for ingested pollutants and toxic substances.

## Author Contributions

S.A., M.A., K.C.N., and C.A.A. conceptualized and designed the study. S.A. conducted the primary investigation with experimental support from H.B., L.C., O.A., C.Z., Y.P., D.Y., X.L., and E.I.B. S.A. and I.O. were responsible for data analysis, visualization, and interpretation of the results, with technical support from C.B. and B.R. C.A.A. and M.A. supervised the project. H.I.A. was responsible for the Raman spectroscopy analysis. N.B.M. and M.E.S. conducted the SEM analysis. S.A., H.B. and C.A.A. wrote the manuscript with critical input from M.A., K.C.N., and R.D. All authors critically reviewed the content and approved the final version for submission.

## Funding

The authors have nothing to report.

## Conflicts of Interest

R.D. is a non‐executive Board Director at Seed Health Inc. I.O. is chair of the EAACI Epithelial Cell Biology Working Group. K.C.N. currently reports Grants from National Institute of Allergy and Infectious Diseases (NIAID), National Heart, Lung, and Blood Institute (NHLBI), National Institute of Environmental Health Sciences (NIEHS); Stock options from Phylaxis and IgGenix; Co‐founder of Latitude, Alladapt, BeforeBrands, and IgGenix; Consultant for Excellergy; National Scientific Committee member at Immune Tolerance Network (ITN). Patents include Granulocyte‐based methods for detecting and monitoring immune system disorders, Methods and Assays for Detecting and Quantifying Pure Subpopulations of White Blood Cells in Immune System Disorders, Biological sample preparation device and methods of using the same, Immune cell activation device and method of using the same, Mixed allergen composition and methods for using the same, Methods of Isolating Allergen Specific Antibodies, Microfluidic devise and diagnostic methods for Basophil Activation, and Methods for detecting heavy metals in biological samples. M.A. has received research grants from the Swiss National Science Foundation, Bern; research grant from Stanford University; Leading House for the Latin American Region, Seed Money Grant. She is the Scientific Advisory Board member of Stanford University Sean Parker Asthma Allergy Center, CA; Advisory Board member of LEO Foundation Skin Immunology Research Center, Copenhagen; and Scientific Co‐Chair of World Allergy Congress (WAC) Istanbul, 2022, Scientific Programme Committee Chair, EAACI. C.A.A. has received research grants from the Swiss National Science Foundation, European Union (EU CURE, EU Syn‐Air‐G), Novartis Research Institutes (Basel, Switzerland), Stanford University (Redwood City, Calif), Seed Health (Boston, USA) and SciBase (Stockholm, Sweden); is the Co‐Chair for EAACI Guidelines on Environmental Science in Allergic diseases and Asthma; is on the Advisory Boards of Sanofi/Regeneron (Bern, Switzerland, New York, USA), Stanford University Sean Parker Asthma Allergy Center (CA, USA), Novartis (Basel, Switzerland), Glaxo Smith Kline (Zurich, Switzerland), Bristol‐Myers Squibb (New York, USA), Seed Health (Boston, USA), and SciBase (Stockholm, Sweden); and is the Editor‐in‐Chief of Allergy. Other authors declare that they have no relevant conflicts of interest.

## Supporting information


**Figure S1:** (A) Representative brightfield (top) and fluorescence microscopy images (bottom) of the PET particles used in the study. The particles were stained with Nile Red to confirm their polymeric nature. Scale bars are 275 μm for 10× magnification and 150 μm for 20× magnification. (B) Time‐course analysis of intracellular Reactive Oxygen Species (ROS) generation in intestinal epithelial cells exposed to various concentrations of PET particles over 24 h. ROS levels are expressed relative to the control group at the start of the experiment. Data points represent the mean ± standard deviation. (C) Scanning electron microscopy (SEM) images of PET microplastics at low and intermediate magnifications (400×, 1000×, and 5000×). The micrographs reveal irregular, fragmented particle morphology with rough and heterogeneous surface topography, consistent with mechanically degraded PET microplastics. Scale bars: 100 μm and 10 μm, as indicated. (D) High‐resolution SEM images (50,000×, 100,000×, and 200,000× magnification) demonstrate the presence of nanoscale fragments associated with the parent PET microplastics. Discrete nanoparticulate structures with diameters of approximately 213.5 nm and 217.3 nm are indicated, providing direct evidence of co‐existing nanoplastic fractions. Scale bars: 1 μm and 300 nm. (E) Optical microscopy image of PET microplastics dispersed on a glass slide, illustrating particle size heterogeneity and irregular shape distribution. The image shows the presence of mixed‐size plastic fragments, which supports the environmentally relevant exposure model used in this study. Scale bar: 200 μm. (F) Raman intensity map at 1614 cm^−1^ overlaid on the optical image, where brighter regions reflect the particles' 3D morphology. (G) Raman spectrum of the particle showing characteristic PET bands, confirming its identity as polyethylene terephthalate.
**Figure S2:** Violin plots show the relative abundance (Normalized Protein eXpression, NPX) of individual proteins secreted into the culture medium after 48 h of exposure to increasing concentrations of PET microplastics. Data were analyzed by a one‐way ANOVA followed by Tukey's Honest Significant Difference (HSD) post hoc test. Asterisks indicate a significant difference compared to the control group (*p.adj* < 0.05, *p.adj < 0.01, **p.adj < 0.001).
**Figure S3:** Dose‐dependent transcriptomic responses following PET microplastic exposure.
(A) Bar plot showing the number of differentially expressed genes (DEGs) across increasing exposure doses, stratified by upregulated and downregulated transcripts. A progressive increase in DEG counts is observed with rising particle concentrations. (B) UpSet plot illustrating the intersection of DEGs across dose groups, demonstrating minimal overlap between low‐ and high‐dose exposures and highlighting dose‐specific transcriptional signatures. (C) Gene set enrichment analysis (GSEA) of significantly dysregulated pathways, including mitochondrial translation, embryonic organ development, actin filament bundle organization, cell–cell adhesion via plasma membrane adhesion molecules, and negative regulation of sprouting angiogenesis. Dot size indicates gene count, and color represents adjusted *p*‐values. (D) Spearman correlation analysis between exposure dose and pathway activity scores, identifying both positive and negatively correlated biological processes, including ferroptosis, signal transduction by p53 class mediators, vitamin transport, and cellular response to heat stress. (E–F) Representative dose–response correlations for selected pathways, including positive chemotaxis, endosome‐to‐lysosome transport, ferroptosis, and glycoprotein metabolic processes, demonstrating graded activation or suppression with increasing exposure levels. (G–H) Expression profiles of oxidative stress‐ and xenobiotic‐response genes (*CYP1A1*, *NQO1*, *SLC7A11*, *TXNRD1*, *HMOX1*, *SQSTM1*, *RELB*, and *GADD45A*) showing strong positive correlations with dose. (I) Negative dose‐dependent expression trends of metabolic and lipid‐related genes (*ACSS1*, *SLC2A5*, *VIL1*, and *DGAT2*), indicating suppression of anabolic pathways at higher exposure concentrations. Correlation coefficients (*ρ*) and false discovery rates (FDR) are indicated within each panel. Exposure doses are presented as log10 (Dose +1).

## Data Availability

The data that support the findings of this study are available from the corresponding author upon reasonable request.

## References

[1] Q. Guan , J. Jiang , Y. Huang , et al., “The Landscape of Micron‐Scale Particles Including Microplastics in Human Enclosed Body Fluids,” Journal of Hazardous Materials 442 (2023): 130138. 10.1016/j.jhazmat.2022.130138 36303360

[2] N. Qian , X. Gao , X. Lang , et al., “Rapid Single‐Particle Chemical Imaging of Nanoplastics by SRS Microscopy,” Proceedings of the National Academy of Sciences of The United States of America 121, no. 3 (2024): e2300582121. 10.1073/pnas.2300582121 PMC10801917 38190543

[3] K. Senathirajah , S. Attwood , G. Bhagwat , M. Carbery , S. Wilson , and T. Palanisami , “Estimation of the Mass of Microplastics Ingested–A Pivotal First Step Towards Human Health Risk Assessment,” Journal of Hazardous Materials 404 (2021): 124004. 10.1016/j.jhazmat.2020.124004 33130380

[4] R. C. Thompson , W. Courtene‐Jones , J. Boucher , S. Pahl , K. Raubenheimer , and A. A. Koelmans , “Twenty Years of Microplastic Pollution Research—What Have We Learned?,” Science 386, no. 6720 (2024): eadl2746. 10.1126/science.adl2746 39298564

[5] Z. Wang , S. Wang , S. Liu , F. Li , Q. Bu , and X. An , “Polystyrene Microplastics Induce Potential Toxicity Through the Gut‐Mammary Axis,” Npj Science of Food 9, no. 1 (2025): 139. 10.1038/s41538-025-00517-5 PMC12259839 40659623

[6] M. Xu , J. Chen , L. Gao , S. Cai , and H. Dong , “Microplastic Exposure Induces HSP90α Secretion and Aggravates Asthmatic Airway Remodeling via PI3K‐Akt‐mTOR Pathway,” Ecotoxicology and Environmental Safety 291 (2025): 117828. 10.1016/j.ecoenv.2025.117828 39923560

[7] C. A. Akdis , “Does the Epithelial Barrier Hypothesis Explain the Increase in Allergy, Autoimmunity and Other Chronic Conditions?,” Nature Reviews Immunology 21, no. 11 (2021): 739–751. 10.1038/s41577-021-00538-7 33846604

