# Supplementary material for: Polyethylene Terephthalate Micro and Nanoplastics Induce Oxidative and Endoplasmic Reticulum Stress, Compromising Intestinal Epithelial Integrity and Viability
Source: Allergy. 2026 Mar 25;81(10):3728–33. doi: 10.1111/all.70291 (PMC13646877; doi:10.1111/all.70291)
Supplement: Supplementary file 1 [file ALL-81-3728-s001.docx]

*Online Supplementary files for*

**Polyethylene Terephthalate Micro- and Nanoplastics Induce Oxidative and Endoplasmic Reticulum Stress, Compromising Intestinal Epithelial Integrity and Viability**

Sena Ardicli^1,2^, Huseyn Babayev^1^, Lihong Chang^1^, Ozge Ardicli^1,3^, Can Zeyneloglu^1^, Yagiz Pat^1^, Duygu Yazici^1^, Xiaoqing Liu^1^, Ezgi Irem Bektas^4^, Carina Beha^1^, Halil I. Akyildiz^5^, Nazrin B. Mammadova^6^, Muhammed Emin Sari^7^, Beate Rückert^1^, Raja Dhir^8^, Ismail Ogulur^1^, Kari C. Nadeau^9^, Mubeccel Akdis^1^, Cezmi A. Akdis^1^

^1^Swiss Institute of Allergy and Asthma Research (SIAF), University of Zurich, Davos, Switzerland

^2^Department of Genetics, Faculty of Veterinary Medicine, Bursa Uludag University, Bursa, Turkiye

^3^Division of Food Processing, Milk and Dairy Products Technology Program, Karacabey Vocational School, Bursa Uludag University, Bursa, Turkiye

^4^AO Research Institute, Davos, Switzerland

^5^Department of Textile Engineering, Faculty of Engineering, Bursa Uludag University, Bursa, Turkiye

^6^Department of Physics, Faculty of Science, Selcuk University, Konya, Turkiye

^7^Department of Medical Biology, Faculty of Medicine, Necmettin Erbakan University, Konya, Turkiye

^8^SEED Inc. Co., Los Angeles, California, USA

^9^Department of Environmental Health, Harvard T.H. Chan School of Public Health, Boston, Massachusetts, USA

**Corresponding author:**

Cezmi A. Akdis, Swiss Institute of Allergy and Asthma Research (SIAF), University of Zurich, Herman-Burchard-Strasse 9, CH-7265 Davos, Wolfgang, Switzerland, e-mail: akdisac@siaf.uzh.ch

A. **Supplementary Materials and Methods**

*1. Generation and characterization of polyethylene terephthalate (PET) particle suspensions*

Food-grade PET beverage bottles were rinsed with analytical-grade water, dried under a laminar flow hood, and manually cut into approximately 5 mm^2^. The fragments were transferred to a stainless-steel ball mill (Retsch CryoMill, Haan, Germany) and ground for three cycles of three minutes at 25 Hz, with intermediate one-minute cooling intervals. The resulting powder was sequentially sieved through stainless-steel meshes to remove particles exceeding 50 micrometers.

The retained fraction was dispersed in endotoxin-free cEMEM and sonicated for 10 min at 40 kHz to disrupt agglomerates. Particle number concentration in each working dilution was quantified in triplicate. The number of PET particles for each condition was quantified using the LUNA-II™ Brightfield Cell Counter (Logos Biosystems, Inc.), which effectively detected and counted the particles. Based on these measurements, PET particles were prepared at doses ranging from approximately 200 to 72,000 particles/mL. The particles were subsequently suspended in complete EMEM (cEMEM) for use in exposure experiments. LPS contamination was assessed using the Lonza Kinetic-QCL™ Kinetic Chromogenic LAL Assay (sensitivity: 0.005 to 50 EU/ml). Only preparations confirmed to be free of LPS contamination were used in cell culture experiments.

For microplastic staining, a 1 mg/mL stock solution of Nile Red (Sigma-Aldrich) was prepared in a solvent mixture of 25% acetone and 75% ethanol. For staining, plastic particles were incubated for 2 hours at 80°C in a solution of 0.1% SDS in water containing a final Nile Red concentration of 20 µg/mL. Following the incubation, the particles were washed twice with a 0.1% SDS solution to remove excess dye. A drop of the particle suspension was then placed on a glass slide for microscopy. Images were acquired using an EVOS M7000 Imaging System in both brightfield and RFP (red fluorescent protein) channels (Figure S1A). This method is suitable for microplastics but cannot accurately quantify or confirm the presence of nanoparticles (<1 µm).

*2. Chemical profiling of PET microplastics*

Chemical characterization of the PET microplastics was carried out using a confocal Raman spectrometer (Renishaw, in Via Qontor) equipped with a Leica DM2700 microscope. The probing laser was selected at 785 nm with a grating of 1200 lines/mm. PET particles were dispersed in DI water, and 3 mL of the dispersion was placed on a metal slide before drying at 50 °C on a hot plate. The Raman spectrum of the particle (Figure S1E-G) shows characteristic PET vibrational bands, including the strong ester C=O stretching at 1727 cm^-1^, aromatic C=C modes at 1614 cm^-1^, the ring-breathing band at 1003 cm^-1^, and the C–O–C/ C–O stretching features at 1295, 1114, and 1102 cm^-1^. Additional PET-specific low-frequency and skeletal modes appear at 857, 705, 632, and 277 cm^-1^. The collective presence and alignment of these bands provide unambiguous confirmation that the analyzed microplastic is “polyethylene terephthalate (PET).” Furthermore, the Raman map (Figure S2b) reveals intensity variations across the PET particles, primarily due to their three-dimensional morphology. Regions appearing red correspond to surface areas closer to the focal plane, where the curved particle geometry enhances laser coupling. Despite this topographical effect, the spectra are consistent across the map, and a simple dimensional assessment indicates particle sizes on the order of tens to a few hundred micrometers.

3. Scanning Electron Microscopy (SEM)

Samples were mounted onto aluminum stubs using double-sided carbon tape. To enhance surface conductivity, the samples were coated with approximately 10 nm of gold using a Cressington 108auto sputter coater, integrated with a Cressington MTM 10 thickness monitor.

Microstructural analysis was performed using a ZEISS EVO LS 10 SEM with a secondary electron detector. Imaging was conducted at an accelerating voltage of 20 kV, with magnifications ranging from 400× to 200,000×.

*3. Organ‑on‑a‑chip platform*

Caco-2 epithelial micro-tissues were established in the OrganoPlate 3-lane 64 microfluidic device (6405-400-B Mimetas BV, Leiden, The Netherlands). This platform is a membrane-free, microfluidic organ-on-chip system that enables the formation of perfused, three-dimensional tissue models under dynamic flow conditions. The OrganoPlate allows precise spatial organization of cells and continuous medium perfusion, thereby closely mimicking physiological shear stress, nutrient gradients, and cell–cell interactions observed in the human intestinal epithelium. Compared to conventional static transwell cultures, this system provides a more physiologically relevant microenvironment, supporting enhanced epithelial differentiation, polarization, and barrier function. As such, it represents a state-of-the-art in vitro model for studying intestinal responses to environmental exposures. An ECM gel (4 mg/mL) was prepared by mixing 5 mg/mL collagen-I (rat tail), 1 M HEPES (pH 7.2–7.5), and 37 g/L NaHCO₃ in an 8:1:1 ratio, kept on ice, and used within 10 min. Then, 2 μL of gel was loaded into each chip inlet, and the OrganoPlate was incubated for 15 min to allow the gel polymerization.

After incubation, 30 μL of HBSS was added to the gel inlets to prevent the gel from drying. The OrganoPlate was returned to the incubator. The following day, 20,000 Caco-2 cells in 2 μL complete EMEM (cEMEM) were seeded into the right perfusion inlets, followed by 50 μL of cEMEM. The plate was placed at a 75° angle and incubated for 3–4 h to allow cell attachment. Once attached, 50 μL of complete EMEM was added to the remaining perfusion inlets and outlets. Perfusion was initiated using the OrganoFlow (Mimetas BV), set to an inclination of 14° with an 8 min interval for bidirectional flow. Medium was refreshed every 2 days using a multichannel pipette. PET particles were applied to the apical side on day 8 post-seeding, once tubule formation was complete. In the untreated group, only medium was added to the apical compartment.

*4. Exposure design and dosimetry*

Micro‑tissues were apically perfused with medium containing 200, 1000, 10,000, 20,000, and 72,000 particles per milliliter. Here, it should be noted that due to the lack of dedicated nanoparticle tracking systems, PET particle concentrations were estimated using a LUNA automated cell counter. PET particles were quantified by adapting the analysis settings to match the typical size range of erythrocytes (6–8 µm), which approximated the dimensions of the particles used in this study. This size-based gating approach enabled the selective enumeration of PET particles while minimizing background noise and the inclusion of debris. Although the device is primarily designed for cellular analysis, the similarity in size enabled an approximate but consistent quantification of PET particles within this defined range. As PET particles differ from cells in terms of morphology and optical properties, some variation in particle counts was observed, particularly at lower doses. Nevertheless, adjusting doses according to directly counted particle numbers within a defined size range (6–8 µm) provided a more accurate and reproducible approach. The strategy we established minimized the impact of sample heterogeneity and enabled a more reliable dose–response assessment. This approach was validated through numerous independent repetitions, demonstrating improved reproducibility and minimizing the impact of sample heterogeneity in dose–response analyses.

Therefore, each target dose is reported with its corresponding measured range as follows:

1) 180-283: 200p/mL
2) 980-1149: 1000p/mL
3) 8754-10109: 10000p/mL
4) 18559-21210: 20000p/mL
5) 71443-72154: 72000p/mL

The particle concentration ranges presented in this study were determined based on multiple independent measurements conducted by the researchers. Given the inherent variability associated with quantifying PET particles using an automated cell counter not specifically designed for non-biological particulates, these ranges reflect the observed fluctuations across replicates. To facilitate clarity and consistency throughout the manuscript, a representative particle concentration was designated for each range and used as a nominal dose in all subsequent analyses and figures.

PET particles are prone to acquiring electrostatic charge due to their dielectric nature and high surface-area-to-volume ratio. This phenomenon is particularly prominent during handling steps involving friction with plasticware, such as pipette tips and microcentrifuge tubes, which can induce triboelectric charging. These behaviors led to inconsistent particle recovery and significant variability in suspension-based dilution protocols.

Despite the use of an electrostatic mat, plastic particles exhibited a strong tendency to aggregate and adhere to pipette tips, leading to inconsistent recovery during conventional suspension dilution procedures. As these challenges resulted in inaccurate estimations of particle concentrations, we observed that dose adjustment based on direct particle counts provided a more reliable and reproducible dose-dependent response. Therefore, dosing was standardized according to the number of particles rather than the volume of the suspension.

Basal channels received particle-free medium to maintain a chemical gradient. Perfusion continued for one, twenty-four, forty-eight, seventy-two, or ninety-six hours under continuous rocking. Vehicle-treated chips served as controls. Each treatment arm consisted of three biological replicates derived from independent passages.

*5. The assessment of epithelial barrier integrity*

*5A. Transepithelial electrical resistance (TEER) measurement in Caco-2 cells cultured on OrganoPlates*

TEER measurements were conducted using the OrganoTEER system (Mimetas, NL) following media refreshment to establish baseline values. Graded particle numbers of PET were applied to the apical side of the chips. TEER was recorded at 24, 48, and 72 hours post-treatment. The impact of each treatment was expressed as a percentage relative to the baseline TEER values. Each condition was tested in quadruplicate.

*5B. Paracellular permeability*

Paracellular permeability was evaluated at every time point by introducing 50 μM Lucifer Yellow into the apical channel and determining its concentration in the basal effluent after 60 min using a fluorescence spectrophotometer set to 420-nm excitation and 535-nm emission. Apparent permeability coefficients were calculated according to the manufacturer’s algorithm. Chips exhibiting technical artefacts, defined as spontaneous leakage of more than ten percent of the apical tracer within ten minutes, were excluded a priori.

*6. Cell viability assay*

Cell viability was assessed using the MTT assay (Invitrogen, USA) according to the manufacturer’s protocol. In brief, 2×10^4^ cells were cultured in 96-well plates and exposed to the experimental conditions. MTT solution was then added, and plates were incubated at 37°C for 4 hours to allow viable cells to convert MTT into formazan crystals. After incubation, the crystals were dissolved in a solubilizing agent, such as DMSO, and the absorbance was recorded at 540–570 nm using a microplate reader to quantify cell viability.

*7. Determination of intracellular reactive oxygen species*

Intracellular reactive oxygen species (ROS) generation was quantified using CM-H_2_DCFDA. Initially, Caco-2 cells were seeded at a concentration of 10,000 cells/well in 96-well plates and incubated overnight in a 37°C humidified incubator with 5% CO_2_. The following day, cells were loaded with 20 µM CM-H2DCFDA for 45 minutes. After the dye incubation, cells were washed and then treated with medium containing PET microplastics at doses of ~200, 1,000, 10,000, 20,000, and 72,000 particles/mL. Vehicle-treated cells served as the negative control, while a 10 mM solution of H_2_O_2_ was used as a positive control for ROS induction. Fluorescence was measured at the beginning of the assay and after one and twenty-four hours of incubation using a microplate reader (Berthold, Germany) with an excitation wavelength of 485 nm and an emission wavelength of 535 nm.

*8. Targeted proteomics for secreted protein profiling*

Basal effluents collected at 48h were clarified by centrifugation at 4,000 g for 5min and analyzed using the Olink immune response and inflammation panels (OLINK Proteomics AB, Uppsala, Sweden), each containing 92 proteins. The Olink-generated data were pre-analyzed and quality-controlled by the platform-specific “Olink NPX manager” software (https://olink.com/). Data were reported as normalized protein expression units on a log2 scale following inter‑plate calibration.

*9. RNA isolation and sequencing*

Total RNA was extracted from replicate chips at 96 hours using the RNeasy Plus Micro kit (Qiagen), quantified with a NanoDrop 2000 Spectrophotometer (Thermo Fisher Scientific), and assessed for integrity on a 2200 TapeStation (Agilent Technologies). Polyadenylated RNA was enriched using oligo-dT magnetic beads, and sequencing libraries were prepared with the TruSeq Stranded mRNA kit according to the manufacturer’s instructions. Paired-end sequencing (150 bp) was performed on an Illumina NovaSeq 6000 system.

*10. Bioinformatics and statistical analysis*

*10A. Quality control and exploratory data analysis*

Read‑level quality metrics, including per‑base sequence quality, GC distribution, and over‑represented sequences, were summarised with FastQC and aggregated in MultiQC. Adapter trimming and quality filtering were executed with BBDuk (BBMap suite). Clean reads were aligned to the *Homo sapiens* GRCh38.p14 reference genome using the Subread package (v2.0.6) with default settings for paired-end sequencing. Transcript features were annotated according to GENCODE Release 46, and gene-level read counts were quantified using featureCounts (Subread package). Only transcripts with a total count greater than ten across all samples and detected in at least three samples were retained for downstream analysis.

*10B. Differential gene expression modelling*

Differential expression analysis was performed using the DESeq2 package (v1.48.1). The analysis employed a generalized linear model with a factor for the treatment condition. Direct pairwise comparisons were made between each particle concentration and the control group. Normalization was conducted using the median of ratios method. Genes were identified as differentially expressed in each comparison if they had an absolute log2 fold change greater than 1 and a Benjamini–Hochberg-adjusted *p*-value below 0.05.

Significantly regulated genes were subjected to overrepresentation analysis against the Gene Ontology Biological Process and KEGG pathway databases using the clusterProfiler (v4.16.0) package.

*11. Proximity extension assay statistical analysis*

Statistical analysis of the Olink Proximity Extension Assay (PEA) data was performed using the ‘OlinkAnalyze’ package (version 4.3.1) in R. Normalized Protein eXpression (NPX) values, which are on a log2 scale, were used as the input for the analysis. To identify differentially expressed proteins across the experimental conditions, an analysis of variance (ANOVA) was conducted for each protein. The resulting p-values were adjusted for multiple comparisons using the Benjamini-Hochberg method to control the false discovery rate (FDR). Following the ANOVA, pairwise comparisons between groups were performed using Tukey's Honest Significant Difference (HSD) post-hoc test. Proteins with an adjusted p-value less than 0.05 were considered statistically significant.

*12. Statistical framework and reproducibility*

Continuous variables were summarized as means with standard deviations unless otherwise indicated. Prior to analysis, data distributions were inspected with Shapiro–Wilk tests and Q–Q plots, and homogeneity of variance was assessed with Levene’s test. As parametric assumptions were satisfied, statistical significance between groups was determined using analysis of variance (ANOVA), followed by Dunnett's post hoc test for multiple comparisons against the control group. Time-course data were analyzed with linear mixed-effects models. All analyses were executed in R (version 4.5.0) or GraphPad Prism.

The raw data and computer code supporting the findings of this study are available from the corresponding author upon reasonable request.

B. **Supplementary figures**

**Figure S1.** **(A)** Representative brightfield (top) and fluorescence microscopy images (bottom) of the PET particles used in the study. The particles were stained with Nile Red to confirm their polymeric nature. Scale bars are 275 µm for 10x magnification and 150 µm for 20x magnification. **(B)** Time-course analysis of intracellular Reactive Oxygen Species (ROS) generation in intestinal epithelial cells exposed to various concentrations of PET particles over 24 hours. ROS levels are expressed relative to the control group at the start of the experiment. Data points represent the mean ± standard deviation. **(C)** Scanning electron microscopy (SEM) images of PET microplastics at low and intermediate magnifications (400×, 1,000×, and 5,000×). The micrographs reveal irregular, fragmented particle morphology with rough and heterogeneous surface topography, consistent with mechanically degraded PET microplastics. Scale bars: 100 µm and 10 µm, as indicated. **(D)** High-resolution SEM images (50,000×, 100,000×, and 200,000× magnification) demonstrate the presence of nanoscale fragments associated with the parent PET microplastics. Discrete nanoparticulate structures with diameters of approximately 213.5 nm and 217.3 nm are indicated, providing direct evidence of co-existing nanoplastic fractions. Scale bars: 1 µm and 300 nm. **(E)** Optical microscopy image of PET microplastics dispersed on a glass slide, illustrating particle size heterogeneity and irregular shape distribution. The image shows the presence of mixed-size plastic fragments, which supports the environmentally relevant exposure model used in this study. Scale bar: 200 µm. **(F)** Raman intensity map at 1614 cm⁻¹ overlaid on the optical image, where brighter regions reflect the particles’ 3D morphology. **(G)** Raman spectrum of the particle showing characteristic PET bands, confirming its identity as polyethylene terephthalate.

**Figure S2.** Violin plots show the relative abundance (Normalized Protein eXpression, NPX) of individual proteins secreted into the culture medium after 48 hours of exposure to increasing concentrations of PET microplastics. Data were analyzed by a one-way ANOVA followed by Tukey's Honest Significant Difference (HSD) post-hoc test. Asterisks indicate a significant difference compared to the control group (*p.adj* < 0.05, **p.adj* < 0.01, ***p.adj* < 0.001).

**Figure S3.** Dose-dependent transcriptomic responses following PET microplastic exposure.

**(A)** Bar plot showing the number of differentially expressed genes (DEGs) across increasing exposure doses, stratified by upregulated and downregulated transcripts. A progressive increase in DEG counts is observed with rising particle concentrations. **(B)** UpSet plot illustrating the intersection of DEGs across dose groups, demonstrating minimal overlap between low- and high-dose exposures and highlighting dose-specific transcriptional signatures. **(C)** Gene set enrichment analysis (GSEA) of significantly dysregulated pathways, including mitochondrial translation, embryonic organ development, actin filament bundle organization, cell–cell adhesion via plasma membrane adhesion molecules, and negative regulation of sprouting angiogenesis. Dot size indicates gene count, and color represents adjusted p-values. **(D)** Spearman correlation analysis between exposure dose and pathway activity scores, identifying both positive and negatively correlated biological processes, including ferroptosis, signal transduction by p53 class mediators, vitamin transport, and cellular response to heat stress. **(E–F)** Representative dose–response correlations for selected pathways, including positive chemotaxis, endosome-to-lysosome transport, ferroptosis, and glycoprotein metabolic processes, demonstrating graded activation or suppression with increasing exposure levels. **(G–H)** Expression profiles of oxidative stress- and xenobiotic-response genes (*CYP1A1*, *NQO1*, *SLC7A11*, *TXNRD1*, *HMOX1*, *SQSTM1*, *RELB*, and *GADD45A*) showing strong positive correlations with dose. **(I)** Negative dose-dependent expression trends of metabolic and lipid-related genes (*ACSS1*, *SLC2A5*, *VIL1*, and *DGAT2*), indicating suppression of anabolic pathways at higher exposure concentrations. Correlation coefficients (ρ) and false discovery rates (FDR) are indicated within each panel. Exposure doses are presented as log10(Dose + 1).

C. **Extended Discussion**

A critical prerequisite for interpreting any microplastic exposure study is the precise physicochemical characterization of the test material, as uncertainties in polymer identity, surface structure, or particle composition can significantly confound biological outcomes. In this context, rigorous validation of the experimental particles was essential to ensure the reliability of all downstream analyses. As shown in Figure S1, the polymeric identity of the particles was rigorously validated using complementary spectroscopic and morphological approaches. Raman spectral analysis (Fig. S1G) revealed characteristic vibrational bands that are diagnostic for PET, including prominent peaks at approximately 1727 cm⁻¹ (C=O stretching of ester groups), 1615 cm⁻¹ (aromatic ring stretching), 1290–1178 cm⁻¹ (C–O stretching), and 1002 cm⁻¹ (benzene ring breathing mode), confirming the chemical fingerprint of PET microplastics. These spectral features are in strong agreement with previously reported Raman signatures of PET, thus excluding potential polymeric contaminants. Spatial Raman mapping (Fig. S1F) further demonstrated homogeneous spectral distribution across the particles, indicating a consistent chemical composition throughout the material.

In parallel, scanning electron microscopy (SEM) provided high-resolution morphological validation (Fig. S1C–D). Low-magnification images (400×–5k×) revealed irregular, fragmented microplastic structures with rough and heterogeneous surfaces, consistent with environmentally relevant patterns of PET degradation. At higher magnifications (50k×–200k×), SEM analysis uncovered the presence of nanoscale debris embedded on and detached from the parent microplastic surface. Notably, discrete nanoparticulate structures with diameters of approximately 213.5 nm and 217.3 nm were directly visualized (Fig. S1D), providing compelling evidence that the PET material contains a substantial nanoplastic fraction. This observation is particularly important, as nanoplastics possess enhanced surface area-to-volume ratios, increased reactivity, and higher biological penetrability compared to microplastics, which may amplify their toxicological impact.

In brief, the combined Raman spectroscopic and SEM analyses conclusively confirm that the experimental material consists of PET microplastics and simultaneously demonstrates the presence of co-existing nanoplastic particles. This multi-scale characterization highlights the structural complexity of the material and strengthens the biological relevance of the exposure model employed in this study.

Our integrated analysis suggests that the toxicological impact of PET microplastics on the intestinal epithelium occurs in a dose-dependent manner. A crucial insight from our data is the distinction between barrier dysfunction in sub-lethal doses and dose-dependent cytotoxicity. The observation that TEER values decline significantly at concentrations that do not yet impact cell viability (e.g., 10,000 particles/mL) implies that even low-level, chronic exposure could progressively undermine epithelial defenses, thereby rendering individuals more susceptible to enteric infections and inflammatory conditions.

The enrichment of the GO term "intrinsic apoptotic signaling pathway associated with endoplasmic reticulum stress" suggests a potential crosstalk between these cellular programs. Oxidative stress is known to disrupt protein folding within the ER, triggering the unfolded protein response (UPR). Persistent UPR activation, marked in our study by the upregulation of DDIT3 (CHOP), can switch the response from pro-survival to pro-apoptotic. The simultaneous upregulation of genes like TP53 indicates that the cells, when faced with overwhelming damage from both oxidative and ER stress, activate programmed cell death pathways. This convergence of stress signals likely explains the sharp drop in viability observed only at the highest exposure levels. Notably, under conditions of persistent or severe stress, particularly when oxidative damage is coupled with ER stress, cells initiate integrated stress responses that may culminate in the activation of apoptotic pathways if homeostasis cannot be restored. Chronic ER stress leads to sustained UPR signaling, which initially aims to restore normal ER function but can shift toward pro-apoptotic signaling cascades upon prolonged activation ^1^. The excessive cellular damage promotes stabilization and activation of TP53, a central regulator of cell fate, thereby tipping the balance toward apoptosis. Together, these mechanisms likely underlie the dramatic decline in cell viability observed at the highest exposure levels, where the combined burden of oxidative and ER stress overwhelms the cellular adaptive capacity for survival ^1,2^.

As illustrated in Figure S3, transcriptomic profiling revealed a clear dose-dependent response to PET microplastic exposure, characterized by a progressive increase in the number of differentially expressed genes (DEGs) across concentrations (Fig. S3A). At lower exposure doses (200–1,000 p/mL), only a limited number of DEGs were detected, indicating that cellular homeostatic mechanisms initially buffer microplastic-induced stress. The restricted transcriptional perturbation at these concentrations suggests the activation of early adaptive responses rather than overt toxicity. This is further supported by the minimal degree of DEG overlap observed between low and high doses in the UpSet intersection analysis (Fig. S3B), implying that distinct transcriptional programs are engaged depending on the exposure intensity.

It is worth noting that the highest exposure dose (72,000 p/mL) resulted in a dramatic transcriptional shift, yielding 5,023 DEGs (Fig. S3B), indicative of a threshold-dependent molecular response. Functional enrichment analysis revealed significant perturbations in pathways associated with mitochondrial translation, embryonic organ development, actin filament bundle organization, and cell–cell adhesion via plasma membrane adhesion molecules (Fig. S3C). Importantly, several of these pathways already showed subtle modulation at intermediate doses, suggesting a gradual transition from compensatory remodeling to pathological dysregulation as exposure increased.

Spearman correlation analysis further highlighted dose–response relationships for multiple biological processes (Fig. S3D–F). While positive chemotaxis exhibited a strong positive correlation with dose (ρ = 0.87, p = 2.2 × 10⁻⁵), the low-dose groups clustered near the baseline, indicating that migratory signaling was preserved at environmentally relevant concentrations. Conversely, endosome-to-lysosome transport and glycoprotein metabolic processes showed significant negative correlations (ρ = –0.81 and –0.94, respectively), with early suppression detectable already at moderate doses, suggesting that intracellular trafficking and post-translational modification pathways are highly sensitive to microplastic exposure. Notably, ferroptosis-related pathways exhibited a progressive positive correlation (ρ = 0.75, p = 0.0012), with the low-dose groups showing minimal activation, supporting a dose-threshold model for iron-dependent lipid peroxidation–mediated cell death (Fig. S3E).

At the gene level, oxidative stress and xenobiotic response genes, such as *CYP1A1*, *NQO1*, *TXNRD1*, *SLC7A11*, *HMOX1*, *SQSTM1*, *RELB*, and *GADD45A*, exhibited a gradual increase in expression beginning at intermediate doses and becoming highly pronounced at the highest concentration (Fig. S3G–H). This graded response indicates a stepwise induction of cytoprotective defense mechanisms that are insufficient to maintain redox balance under high exposure conditions. In contrast, metabolic regulators, including *ACSS1*, *SLC2A5*, *VIL1*, and *DGAT2*, exhibited progressive downregulation with increasing dose (Fig. S3I), while remaining largely stable at low doses, suggesting that metabolic suppression is a late-stage event that occurs only after cellular compensation has failed.

Collectively, the multi-layered analyses demonstrate that PET microplastic exposure induces a biphasic molecular response (Figure S3), characterized by adaptive transcriptional buffering at low doses and extensive pathway dysregulation at higher concentrations. This dose-dependent shift from homeostatic compensation to pathological remodeling provides critical mechanistic insight into microplastic toxicity, highlighting the importance of considering sub-toxic exposure levels in environmental risk assessment.

From a toxicological perspective, these data raise vital questions regarding the threshold levels at which microplastics incite clinically significant harm in the intestinal epithelium. While the *in vitro* doses deployed here may exceed typical environmental exposures, the pervasive nature of plastic in marine and terrestrial ecosystems underscores the potential for tissue accumulation of microplastics, particularly in high-risk populations or those with compromised gut integrity. This work provides further support for the development of strategies and policies aimed at mitigating the cellular and overall health effects of microplastic contamination in the environment and the human food chain.

*Limitations of the study*

While our findings provide valuable insights into the cellular and molecular responses of intestinal epithelial cells to PET microplastic exposure, the study has several important limitations that should be acknowledged.

This study was conducted using an *in vitro* model with acute, high-dose exposures. Although these conditions are effective for elucidating mechanisms of toxicity, the relatively higher concentrations used (more than 1000 particles per ml) may not be representative of typical chronic, low-level human exposure through diet. Therefore, caution is warranted when extrapolating these findings to predict *in vivo* outcomes in the general population. However, the cumulative doses in the tissues may exceed some of the doses studied in the present study. The cumulative effects of long-term, low-dose exposure remain an important area for future investigation.

The present study represents exposure to environmentally relevant, mixed-size plastic particles, reflecting realistic scenarios of contamination. The PET microplastics were generated from food-grade beverage bottles, and their polymeric identity was rigorously confirmed by Raman spectroscopy and SEM analysis (Figure S1), ensuring that the experimental material was indeed “polyethylene terephthalate.” SEM imaging further demonstrated the presence of nanoscale fragments associated with the parent microplastics, highlighting the structural complexity of the exposure material. Nevertheless, although the polymer identity was verified, commercial PET products inherently contain a complex mixture of additives, including plasticizers, stabilizers, and colorants, which were not chemically profiled in this study. Therefore, it remains plausible that a fraction of the observed biological effects may arise from the leaching of these additives rather than from the PET polymer backbone alone. A limitation of the current design is the absence of a leachate-only control (supernatant of particle suspensions), which would have enabled discrimination between particle-driven and chemically mediated toxicity. However, it is important to emphasize that Raman spectroscopy clearly showed that the material used was predominantly PET. This was further validated by direct comparison with previously published Raman spectra of PET samples ^3^, confirming a very high level of chemical identity.

As detailed in the supplementary methods, quantifying particle dose posed significant technical challenges. The use of an automated cell counter for non-biological particles and issues with electrostatic aggregation led to variability in the administered dose. Although we standardized dosing based on direct particle counts to mitigate this, the reported concentrations should be considered estimates. This highlights the urgent need for standardized and validated methods for microplastic dosimetry in toxicological research.

Various quantification and validation techniques have been employed in previous studies to determine the number of particles in different water sources and bottled water products. However, detecting and characterizing these particles remains technically challenging. This is particularly evident from the substantial variability observed not only within individual sample sets but also across different studies, highlighting the complexity of standardizing methodologies in this field ^4-6^.

The issue of microplastic pollution has gained increasing attention in recent years, particularly within freshwater sources. In parallel with growing concerns about their ecological and health impacts, substantial efforts have been devoted to developing reliable methods for detecting and characterizing microplastics in environmental matrices. While the analysis of larger microplastic particles poses its own challenges, the detection of smaller particles (especially those measuring less than a micrometer in diameter) has emerged as a particularly complex task due to their invisibility to the naked eye and their diverse physicochemical properties ^6,7^.

In the present study, our primary aim was to draw attention to the dose-dependent cytotoxic potential of PET microplastics on epithelial cells and establish proteomics and transcriptomics platforms that can be used in future studies. By demonstrating clear biological responses , we aim to provide a foundation for future studies that incorporate state-of-the-art analytical platforms and more comprehensive approaches. Such investigations will be essential to fully elucidate the physicochemical properties of microplastics and their implications for human and environmental health.

D. **Supplementary References**

1.	Wang M, Kaufman RJ. Protein misfolding in the endoplasmic reticulum as a conduit to human disease. *Nature*. 2016;529(7586):326-335.

2.	Vousden KH, Prives C. Blinded by the light: the growing complexity of p53. *Cell*. 2009;137(3):413-431.

3.	Peñalver R, Zapata F, Arroyo‐Manzanares N, López‐García I, Viñas P. Raman spectroscopic strategy for the discrimination of recycled polyethylene terephthalate in water bottles. *Journal of Raman Spectroscopy*. 2023;54(1):107-112.

4.	Oßmann BE, Sarau G, Holtmannspötter H, Pischetsrieder M, Christiansen SH, Dicke W. Small-sized microplastics and pigmented particles in bottled mineral water. *Water research*. 2018;141:307-316.

5.	Qian N, Gao X, Lang X, et al. Rapid single-particle chemical imaging of nanoplastics by SRS microscopy. *Proceedings of the National Academy of Sciences*. 2024;121(3):e2300582121.

6.	Surucu O. Electrochemical analysis of PET microplastics and detection of elements in PET bottles. *Microchemical Journal*. 2025;208:112422.

7.	Jung S, Cho S-H, Kim K-H, Kwon EE. Progress in quantitative analysis of microplastics in the environment: A review. *Chemical Engineering Journal*. 2021;422:130154.
